# MIKB: A manually curated and comprehensive knowledge base for myocardial infarction

**DOI:** 10.1016/j.csbj.2021.11.011

**Published:** 2021-11-16

**Authors:** Chaoying Zhan, Yingbo Zhang, Xingyun Liu, Rongrong Wu, Ke Zhang, Wenjing Shi, Li Shen, Ke Shen, Xuemeng Fan, Fei Ye, Bairong Shen

**Affiliations:** a Institutes for Systems Genetics, Frontiers Science Center for Disease-related Molecular Network, West China Hospital, Sichuan University, Sichuan 610212, China; b Tropical Crops Genetic Resources Institute, Chinese Academy of Tropical Agricultural Sciences, Haikou 571101, China

**Keywords:** Myocardial infarction, Knowledge base, Risk factor, Genetics, Multi-omics

## Abstract

Myocardial infarction knowledge base (MIKB; http://www.sysbio.org.cn/mikb/; latest update: December 31, 2020) is an open-access and manually curated database dedicated to integrating knowledge about MI to improve the efficiency of translational MI research. MIKB is an updated and expanded version of our previous MI Risk Knowledge Base (MIRKB), which integrated MI-related risk factors and risk models for providing help in risk assessment or diagnostic prediction of MI. The updated MIRKB includes 9701 records with 2054 single factors, 209 combined factors, 243 risk models, 37 MI subtypes and 3406 interactions between single factors and MIs collected from 4817 research articles. The expanded functional module, *i.e.* MIGD, is a database including not only MI associated genetic variants, but also the other multi-omics factors and the annotations for their functional alterations. The goal of MIGD is to provide a multi-omics level understanding of the molecular pathogenesis of MI. MIGD includes 1782 omics factors, 28 MI subtypes and 2347 omics factor-MI interactions as well as 1253 genes and 6 chromosomal alterations collected from 2647 research articles. The functions of MI associated genes and their interaction with drugs were analyzed. MIKB will be continuously updated and optimized to provide precision and comprehensive knowledge for the study of heterogeneous and personalized MI.

## Graphical abstract

**Figure:**
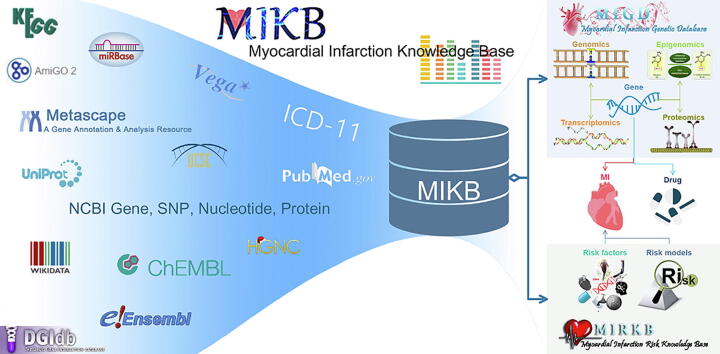

## 1 Introduction

Myocardial infarction (MI), caused by persistent coronary ischemia or hypoxia, is one of the leading causes of death worldwide, resulting in irreversible myocardial necrosis [1]. With increasing attention to MI and the development of modern biological technology, more and more genetic factors and non-genetic factors have been revealed to be related to the occurrence and development of MI [2], [3], [4], [5], [6]. However, its etiology is still not well understood. Generally speaking, with the increase in MI-related literature, people gradually deepen their understanding of MI, and the prevalence and mortality of MI should gradually decrease, but the prevalence and mortality of MI continue to increase in fact [7]. Because knowledge of these articles is scattered, the increasing number of articles makes the understanding of the heterogeneity of MI even more difficult [8]. To help the researchers or clinicians obtain a systematic perspective on MI, we therefore established the first knowledge base for MI to integrate MI-related risk factors and risk models in 2019, named MI Risk Knowledge Base (MIRKB; previous link: http://www.sysbio.org.cn/mirkb/) [9].

In the first version of MIRKB (updated to July 5, 2019), including 8436 records with 1847 single factors, 157 combined factors and 174 risk models collected from 4366 research articles. We divided single factors into molecular factors, imaging factors, physiological factors, clinical factors, environmental factors, lifestyle factors and psychosocial factors based on their characteristics. In this version of MIRKB, we first summarized five methods to classify MI based on articles and ICD-11 (International Classification of Diseases 11th Revision; https://icd.who.int/en), including disease phase, lesion range, infarction location, electrocardiogram (ECG) expression and clinical type. The risk factors, clinical manifestations, prognosis and treatment of different MI subtypes are heterogeneous. Therefore, disease classification is of great significance to the diagnosis, treatment and prognosis of MI. MIRKB 1.1 (updated to December 31, 2019) contained 8738 records with 1924 single factors, 163 combined factors and 197 risk models collected from 4504 research articles. Compared with MIRKB 1.0, MIRKB 1.1 has added 302 records collected from 138 new articles in less than half a year.

The MI Knowledge Base (MIKB; http://www.sysbio.org.cn/mikb/) is an update and expansion of the MIRKB. MIRKB is updated and remains as one of the modules of MIKB. As we all know, MI is not a genetic disease, but it has been proved to be a polygenic disease related to the environment. It is critically important to explore the molecular pathogenesis involved in MI and identify biomarkers for diagnosis, prognosis and treatment. In recent years, with the rapid development of high-throughput sequencing technology, multi-omics data, that is, datasets containing various types of high-dimensional molecular variables (including genomics, epigenomics, transcriptomics, proteomics, metabolomics, *etc.*), are increasingly used in the research of various diseases [10], [11], [12], [13], and no exception for cardiovascular disease (CVD) [14]. Therefore, we added a new genetic module in MIKB for collecting genes from the multi-omics data which are research-supported to be related to MI in humans, named MI Genetic Database (MIGD). We believe that it is of great significance to build this new module to explore the molecular pathogenesis of MI.

## 2 Database content

Currently, MIKB consists of two modules, including MIRKB (http://www.sysbio.org.cn/mikb/mirkb/) and a new genetic module (MIGD; http://www.sysbio.org.cn/mikb/migd/). MIRKB (latest update: December 31, 2020) is a comprehensive database for collecting MI-related risk factors and risk models, while MIGD (latest update: December 31, 2020) is a genetic database for integrating MI-related genes from the multi-omics data. The description of these two modules is as follows.

### 2.1 MIRKB update

**Fig. 1:**
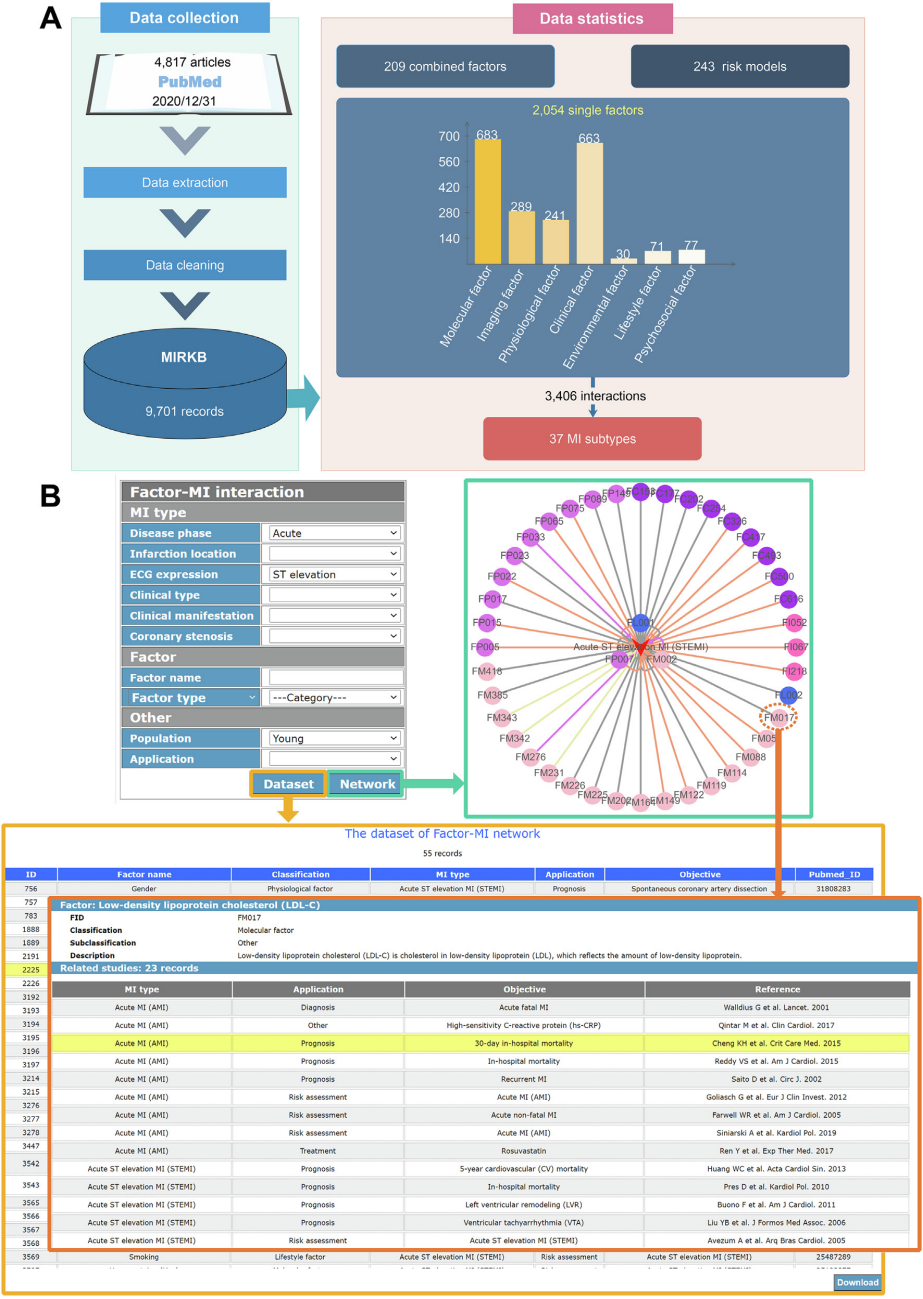
Data collection and ‘Tool’ page of the updated MIRKB.

We updated the data from January 1, 2020 to December 31, 2020. The method of data collection for MIRKB was described in our previous work [9], and the updated MIRKB contains 9701 records with 2054 single factors, 209 combined factors, 243 risk models, 37 MI subtypes and 3406 interactions between single factors and MI collected from 4817 research articles. And the single factors include 683 module factors, 289 imaging factors, 241 physiological factors, 663 clinical factors, 30 environment factors, 71 lifestyle factors and 77 psychosocial factors (Fig. 1A). The statistics for the database can be downloaded from the online page (http://www.sysbio.org.cn/mikb/mirkb/index.html).

The updated MIRKB is slightly different from the previous ones. (1) In the previous versions of MIRKB, the classification of MI was based on disease phase, lesion range, infarction location, ECG expression and clinical type. According to lesion range of MI, MIs are divided into transmural MI and subendocardial MI; based on ECG expression, MIs are divided into ST elevation MI (STEMI) and non-ST elevation MI (NSTEMI). However, transmural MI and STEMI are the same concept, and NSTEMI is also called subendocardial MI. Therefore, we unified the concepts related to these two classification methods and dropped the classification method based on lesion range in this update. Additionally, we added two new classification methods to the MI description, named clinical manifestation and coronary stenosis. According to the clinical manifestation of MI, we divided MI into silent MI and clinical MI. Based on the degree of epicardial coronary stenosis, we added a new MI subtype - MI with non-obstructive coronary arteries (MINOCA). The description of different MI subtypes according to these six classification methods can be obtained from the ‘MI Introduction’ page in the MIKB (http://www.sysbio.org.cn/mikb/MI Introduction.html). (2) The updated MIRKB is more standardized than the previous ones. In the updated MIRKB, the factor names were annotated and standardized by NCBI Gene (https://www.ncbi.nlm.nih.gov/gene) [15], NCBI Protein (https://www.ncbi.nlm.nih.gov/protein) [15], miRBase (http://www.mirbase.org/) [16], ICD-11, Wikipedia (www.wikipedia.org) and scientific articles. (3) The web framework of MIRKB has been slightly changed, and the ‘MI Introduction’ page was replaced with the ‘Tool’ page. The tables on web-pages are improved and more user-friendly than before. Additionally, we added an online button for users to download these tables. (4) The previous ‘Tool’ page was used to calculate the distribution of single factors of a type of MI according to factor classification. In the new version of the ‘Tool’ page, we used Cytoscape.js [17] (https://js.cytoscape.org/) to construct a factor-MI interaction network. As shown in Fig. 1B, users can obtain a factor-MI network based on the MI type, factor name, factor type, research population and factor application that they are interested in. In factor-MI network, arrow nodes and circular nodes represent MIs and factors, respectively; the different colors of circular nodes represent different factor types, including module factor, imaging factor, physiological factor, clinical factor, environment factor, lifestyle factor and psychosocial factor; the different colors of edges represent different factor applications, including risk assessment, diagnostic, prognostic or treatment prediction, *etc.* Users can click on a node or edge in the network to get information about that node or edge. Furthermore, users can get the dataset of the factor-MI network by clicking the ‘Dataset’ button.

### 2.2 The expansion module - MIGD

**Fig. 2:**
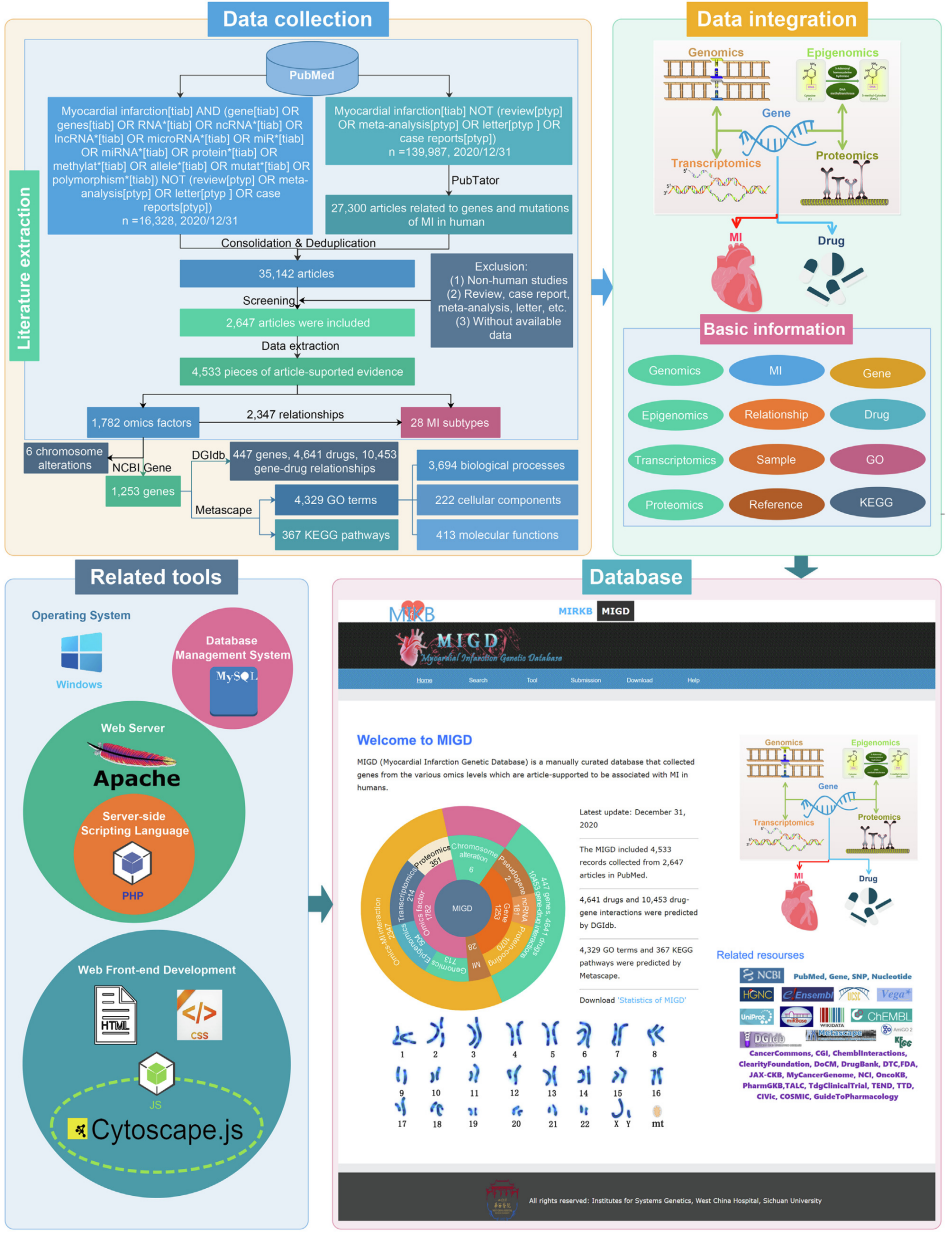
The workflow for the construction of the MIGD.

The workflow for the development of the MIGD is outlined in Fig. 2, including data collection, data integration and annotation, and web platform construction for the database.

#### 2.2.1 Data collection

*Literature extraction:* As shown in Fig. 2, we retrieved the articles in PubMed based on the following two methods, including keyword search and using a text-mining tool (PubTator; https://www.ncbi.nlm.nih.gov/research/pubtator/), and then integrated the articles retrieved by these two methods. In the first, the search keywords were ‘(myocardial infarction[title/abstract] and (gene[title/abstract] or genes[title/abstract] or RNA*[title/abstract] or ncRNA*[title/abstract] or lncRNA*[title/abstract] or microRNA*[title/abstract] or miR*[title/abstract] or miRNA*[title/abstract] or protein*[title/abstract] or methylat*[title/abstract] or allele*[title/abstract] or mutat*[title/abstract] or polymorphism*[title/abstract]) not (review[publication type] or meta-analysis[publication type] or letter[publication type] or case reports[publication type]))’. In the second method, we first used ‘myocardial infarction’ as keywords to search articles; the retrieved articles were then annotated by PubTator; finally, the articles were selected based on their association with MI in humans. The annotation was then performed based on the following criteria.

(1) The retrieval deadline is set as December 31, 2020; (2) Researches are associated with genetic variations, epigenetic signals, transcription and protein expression levels of MI; (3) Omics factors are significantly associated with the risk, diagnosis, prognosis and treatment of MI by experiments. We excluded duplicate studies, animal studies, reviews, case reports, letters and studies without available data.

*GO terms and KEGG pathways:* Metascape [18] (https://metascape.org/) is a powerful tool for gene function annotation that integrates more than 40 authoritative data resources such as gene ontology (GO), KEGG (Kyoto Encyclopedia of Genes and Genomes), Uniprot, *etc*. The Metascape v3.5.20211101 was used to perform GO and KEGG pathway enrichment analyses for MI-related genes at different omics levels*.* In the analyses, the GO terms and KEGG pathways with the number of MI-related genes ≥ 3, *p*-value < 0.05, and enrichment factor ≥ 1.5 were included in the database.

*Gene-drug interactions:* Drug-Gene Interaction Database [19] (DGIdb; https://dgidb.genome.wustl.edu/) is a comprehensive database that integrates many resources, such as DrugBank, PharmGKB, *etc.*, for predicting the interaction between drugs and genes. DGIdb v4.2.0 was therefore used to predict the interaction between MI-related genes and drugs.

#### 2.2.2 Data annotation and integration

*Omics factor:* We divided omics factors into variations at different levels, genomics epigenomics, transcriptomics and proteomics, *etc*. (1) The genomic variations were divided into SNP (Single Nucleotide Polymorphism), CNV (Copy Number Variation) and others. SNPs were annotated according to the reference SNP (rs) IDs of NCBI dbSNP (e.g., rs1010) [15], the CNV and other variations were annotated according to the Human Genome Variation Society (HGVS; http://www.hgvs.org/) sequence variation nomenclature (e.g., NC_000006.12:g.13574131C > A) [20]. However, some of them cannot be annotated by HGVS sequence variation nomenclature due to lack of the key information (e.g., mutation location, mutation type, mutation length, *etc*.), and these were then recorded according to the description of articles (e.g., the SacI polymorphism in the APOC3 gene was recorded as ‘APOC3_SacI’). NCBI dbSNP rs IDs were provided for SNPs, and NCBI Reference Sequence (RefSeq) IDs were provided for gene sequences [15]. (2) The epigenomics data includes DNA methylation signals, and the positions of DNA methylation were annotated based on the description in the reference. (3) The transcriptomics data were divided into expression at messenger RNA (mRNA), microRNA (miRNA), and long non-coding RNA (lncRNA) levels. The mRNAs and lncRNAs were recorded according to the official symbols of the NCBI Gene database [15], while the miRNAs were annotated by the miRbase database [16]. (4) The proteomics data includes expression at protein level, and the proteins were recorded according to Uniprot [21]. The expression changes of RNAs and proteins were recorded as decrease or increase (e.g., the protein levels were significantly increased in MI patients, which was recorded as ‘MI: increased the protein levels’). The application of omics factors was annotated as risk assessment, diagnostic, prognostic or treatment subtyping, *etc.* according to the references. Furthermore, the information of statistics and description on the relationships between omics factors and MIs was also recorded.

*Sample:* The sample information was annotated based on the research descriptions, including sample population (young/elderly), sample size, gender, region, race, medical history, research method, etc.

*Gene:* We annotated the genes related to those omics factors according to NCBI Gene. The information included are the symbol, full name, gene type, chromosome, alias symbols, and gene family. The links of NCBI Gene, Human Gene Nomenclature (HGNC) [22], Ensembl [23], UCSC [24] and Vega [25] were provided for the collected genes. Furthermore, the links of GO [26] and KEGG [27] were provided for GO terms and KEGG pathways involved in MI-related genes, respectively. We also provided links to Wikidata [28] and ChEMBL [29] for gene-related drugs.

*Others:* As mentioned above, we used six methods to classify MI. Otherwise, we provided the PubMed ID, first author name, journal, and publication year of the studies.

#### 2.2.3 Statistics for MIGD

For the construction of the expansion module of MIGD, as shown in Fig. 2, we obtained a total of 35,142 articles by the keywords search (16328 articles) and annotation of PubTator (27300 articles) after the duplicate articles removed. 2647 articles were screened based on inclusion and exclusion criteria, and 4533 records were manually extracted from these articles. We obtained 1782 omics factors, 28 MI subtypes and 2347 factor-MI relationships from those records. According to articles and NCBI Gene, we extracted 1253 genes and 6 chromosomal alterations from omics factors. Furthermore, 10,453 gene-drug relationships between 447 MI-related genes and 4641 drugs were predicted by DGIdb, and 4329 GO terms and 367 KEGG pathways were predicted by Metascape. The GO terms include 3694 biological processes, 222 cellular components and 413 molecular functions.

**Fig. 3:**
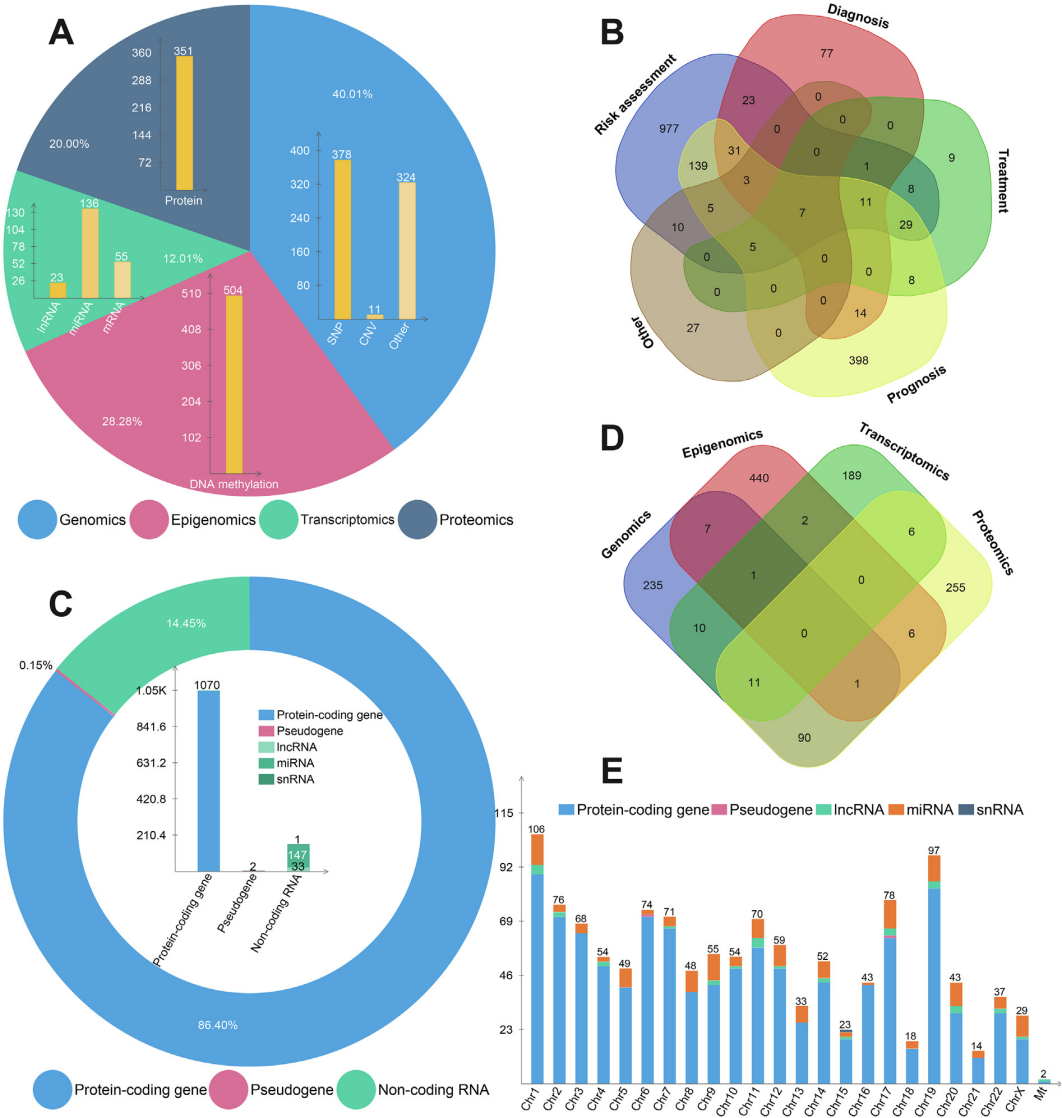
Data statistics in the MIGD database.

*Omics factors:* 1782 MI-related omics factors include 713 genetic variations, 504 DNA methylation signals, 214 RNAs and 351 proteins. Genetic variations include 378 SNPs, 11 CNVs and 324 other variations, while RNAs include 23 lncRNAs, 136 miRNA and 55 mRNA (Fig. 3A). Most of the omics factors are associated with MI risk assessment, followed by prognostic prediction. 18 of the factors are associated with both the risk assessment and personalized medicine of MI (Fig. 3B).

*Genes:* According to Fig. 3C, MIGD includes 1070 protein-coding genes, 2 pseudogenes, 33 lncRNAs, 147 miRNAs and 1 snRNA. Most of the MI associated genes are collected from epigenomic data, follow by proteomic data, 134 genes could be identified from at least two omics data types (Fig. 3D). Human chromosome 1 has the most of MI associated genes compared with other chromosomes, and no MI associated genes reported happen in human chromosome Y (Fig. 3E). The statistics for MIGD can be downloaded from the online page at http://www.sysbio.org.cn/mikb/migd/index.html.

*GO terms and KEGG pathways*: 4329 GO terms and 367 KEGG pathways were significantly enriched for MI-related genes. The details of the significant GO terms and KEGG pathways can be downloaded in MIGD (http://www.sysbio.org.cn/mikb/migd/download.html). In the multi-omics analysis, we screened out GO terms and KEGG pathways that were significantly enriched with MI genes, a total of 314 overlapping GO terms and 4 overlapping KEGG pathways were identified. Table 1 and Table 2 show the top MI-gene enriched GO terms and pathways by LogP values, respectively. The GO terms include biological processes related to cardiovascular system development, blood circulation, response to wounding and positive regulation of locomotion, *etc*. The pathways are ‘pathways in cancer’, ‘hypoxia-inducible factor 1 (HIF-1) signaling pathway’, ‘insulin resistance’ and ‘transforming growth factor (TGF)-beta signaling pathway’.

**Table 1:** Top 10 overlapping GO terms by LogP values in the multi-omics analysis.

GO term	Description	Category	LogP	Number Of Genes
GO:0001568	blood vessel development	biological process	−9.51E + 01	191
GO:0048514	blood vessel morphogenesis	biological process	−9.32E + 01	180
GO:0001525	angiogenesis	biological process	−9.26E + 01	168
GO:0008015	blood circulation	biological process	−8.25E + 01	148
GO:0003013	circulatory system process	biological process	−8.10E + 01	157
GO:0009611	response to wounding	biological process	−7.26E + 01	89
GO:0040017	positive regulation of locomotion	biological process	−7.07E + 01	146
GO:2000147	positive regulation of cell motility	biological process	−6.85E + 01	142
GO:0042060	wound healing	biological process	−6.79E + 01	78
GO:0030335	positive regulation of cell migration	biological process	−6.75E + 01	138

**Table 2:** Overlapping KEGG pathways in the multi-omics analysis.

Pathway	Description	LogP	Number Of Genes
hsa05200	pathways in cancer	−3.14E + 01	96
hsa04066	HIF-1 signaling pathway	−2.04E + 01	23
hsa04931	insulin resistance	−8.22E + 00	21
hsa04350	TGF-beta signaling pathway	−5.95E + 00	16

#### 2.2.4 Web application

A freely available website has been developed to access MIKB , it includes six online pages, *i.e.* ‘Home’, ‘Search’, ‘Tool’, ‘Submission’, ‘Download’, and ‘Help’ pages as shown in Fig. 1.

*Home page*: Users can browse MIGD by clicking the figures of database architecture and chromosome. Moreover, this page provides a brief statistics of the MIGD and links to related databases (e.g., NCBI PubMed, HGNC, UniProt, miRBase, *etc.*). The statistics of MIGD is also available for download (Fig. 1).

**Fig. 4:**
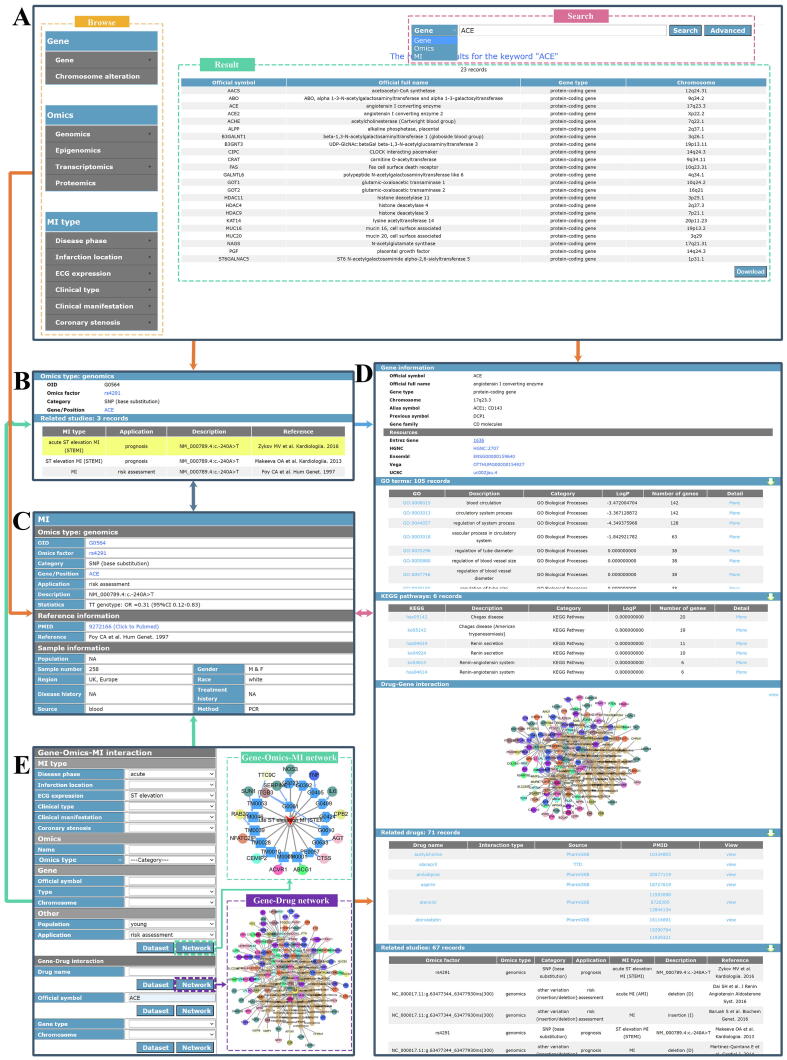
MIGD interface.

*Search page:* As shown in Fig. 4A, the ‘Search’ page includes a navigation bar, a search box and a result box. Users can browse the data in the MIGD according to types of genes, omics factors and MIs. A search box provides three types of search keywords for users, including the names of omics factors, genes or MIs. The results of user browsing and searching are displayed in the result box in the form of a table. If users need more precise query results, the ‘Advanced’ button can be clicked and then the combinatorial search will be provided. The ‘Omic Factor’ and the ‘Gene’ pages (Fig. 4B, Fig. 4D) provide the basic information and related studies. The ‘Omics Factor-MI’ page (Fig. 4C) provides the description of a omics factor-MI interaction, reference information and sample information. Furthermore, MIGD also provides the information about related GO terms and KEGG pathways.

*Tool page:* The interactions between the factors or the genes and drugs could be visualized (Fig. 4E). The network and dataset could be also obtained there. In the gene-omic factor-MI network, the circular nodes, pentagon nodes, and triangular nodes represent genes, omics factors, and MI subtypes respectively; the different colors of the edges between omics factors and MI subtypes represent the potential applications of the omics factors. In the gene-drug network, the circular and hexagon nodes represent genes and drugs respectively. The different colors of the circular nodes in the networks represent the chromosomes of where the genes are located. Users can obtain the information of the omics factors (Fig. 4B), MIs, genes (Fig. 4C), drugs, omics factor-MI interactions (Fig. 4C) and gene-drugs interactions by clicking the dataset table, nodes or edges in the networks.

*Other pages*: Users can contribute to MIGD by submitting related data on the ‘Submission’ page, and download all data in the MIGD on the ‘Download’ page. ‘Help’ page provides users with a usage description file of the MIGD.

## 3 Discussion

The MIKB is now available at http://www.sysbio.org.cn/mikb/, which was expanded and updated based on MIRKB. Currently, MIRKB is a functional module of MIKB, updated to December 31, 2020, including 2054 single factors, 209 combined factors, and 243 risk models. In this version of MIRKB, the web interface was improved and the data annotation was standardized. The Cytoscape.js was used to build the ‘Tool’ page of MIRKB, for visualization of network between risk factors and MI. In the factor-MI network, users can easily obtain the information about the risk factor and its applications in MI medicine by the colors of the edges.

Distinguishing the MI subtypes is a first and vital step for precision medicine of MI. For example, the management and prognosis of NSTEMI patients are different from STEMI patients. The main management of NSTEMI patients includes medical management or ischemia-guided therapy. Invasive coronary intervention is recommended only if the patient has life-threatening high-risk features. But early percutaneous coronary intervention (PCI) is the main treatment method in STEMI patients [30]. A number of studies have found that STEMI patients have a higher risk of short-term or long-term adverse events than NSTEMI patients [31], [32], [33]. Therefore, we used the five methods to describe the MI subtypes, including disease phase, lesion range, infarction location, ECG expression and clinical type in the first version of MIRKB. But we dropped the classification method based on lesion range because it has the same concepts as the method based on ECG expression in this updated version. Furthermore, we added two new methods to classify MI based on clinical manifestation and coronary stenosis of MI. The clinical manifestation of MI varies widely [34], [35]. Patients with clinical MI usually have typical symptoms, changes in ECG, and/or alteration in cardiac biomarkers for personalized treatment and management [36]. Conversely, some patients with silent MI have no or mild symptoms often do not seek medical attention [37]. Studies have shown that silent MI accounts for about half of all MIs [34], [38], [39], [40], and the outcome of these patients is similar to or even worse than that of patients with clinical MI [37], [40], [41], [42], [43]. Therefore, clinical manifestation is an important method for classifying MI to promote the understanding of the difference between clinical MI and silent MI, which helps to improve the diagnosis of silent MI. MINOCA is a special type of MI, which refers to MI without angiographically evident obstructive coronary artery disease (epicardial coronary stenosis ≥ 50%)[31]. The prevalence of MINOCA is estimated to be 5%-14% among patients with MI[44]. Compared with typical MI patients (MI with obstructive coronary artery disease, MICAD), MINOCA patients are more likely to be young, female, STEMI patients, and have fewer cardiovascular risk factors [45]. Many studies have shown that the short-term or long-term prognosis of MINOCA patients is better than those with MICAD, but worse than the general population [46], [47]. It is vital to distinguish MINOCA patients from MICAD patients since they have different MI pathogenesis, prognosis and treatment. Eventually, the new version includes six classification methods of MI, including disease phase, infarction location, ECG expression, clinical type, clinical manifestation and coronary stenosis.

The increasing availability of multi-omics data enables scientists to investigate the molecular mechanism of many complex traits and identify the key players in complex disease [48]. In the MIKB, we built the ‘MIGD’ module for integrating MI-related genes from various omics levels based on the manual collection. MIGD contains 1782 omics factors, 28 MI subtypes, 2347 omics factor-MI interactions, 1253 genes and 6 chromosomal alterations, updated to December 31, 2020. Furthermore, we used DGIdb to predict interactions between MI-genes and drugs. 10,453 interactions between 447 genes and 4641 drugs are included in MIGD. We also used Metascape to perform GO and KEGG pathway enrichment analysis on MI-related genes at the multi-omics levels. The pathways enriched with MI-gene are found related to cardiovascular system development, blood circulation, response to wounding, positive regulation of locomotion, cancer, HIF-1 signaling pathway, insulin resistance and TGF-beta signaling pathway. It is clear that MI is a cardiovascular disease, and the repair process of myocardial tissue after MI is completed by scar repair. In addition to the early occurrence of cardiomyocyte apoptosis, necrosis and inflammation, cell proliferation and migration play a vital role in the repair process of myocardial tissue [49]. Many studies have shown that cancer is associated with increased risk and poor prognosis of MI [50], [51], [52]. Additionally, some studies have shown that patients with MI are at higher risk of cancer than those without MI [53], [54]. Hypoxia-inducible factor-1 (HIF-1) is an important regulatory factor responsible for inducing and promoting the adaptation and survival of cells and entire organisms from normoxia to hypoxia. HIF-1 is a heterodimer composed of HIF-1α and HIF-1β. Hypoxia is one of the main driving factors for metabolic changes in MI. HIF-1 activity has been shown to increase early after MI in ventricular biopsies of patients undergoing coronary artery bypass surgery [55]. Furthermore, HIF-1α overexpressed exosomes rescued the angiogenesis, migration and proliferation of hypoxia-injured human umbilical vein endothelial cells in the rat MI model. It can preserve heart function by promoting the formation of new blood vessels and inhibiting fibrosis. Therefore, HIF-1 may be a potential therapeutic target for MI [56]. According to existing research, insulin resistance is considered to be an important mechanism for the pathogenesis of MI, which is related to atherosclerosis and cardiovascular risk [57], [58]. Ellmers *et al*. found that the blockade of the TGF-beta signaling pathway can lead to a significant improvement in deleterious cardiac remodeling after MI [59].

MIGD as a new module improved our previous knowledgebase as follows. (1) MIGD is a manual curated and highly reliable database. To ensure MIGD is as complete and reliable as possible, two methods (including using keywords and PubTator) were applied to retrieve the literature and collect the data manually. Furthermore, we used NCBI Gene, dbSNP, Nucleotide, miRBase, UniProt, *etc.* to annotate genes and omics factors. (2) MIGD is a genetic database with multi-omics data and provides researchers a systematic perspective on the molecular mechanism of MI. Most of the genetic databases only contain genetic variation data [60], [61], [62], but MIGD includes also multi-omics data, gene-drug interactions, GO terms and KEGG pathways for MI studies. (3) MIGD provides users with a network visualization tool to show the gene-omics factor-MI interactions and gene-drug interactions.

MIKB will be useful to translational research and personalized medicine for MI in several ways. (1) MIKB is a manually curated and comprehensive resource for MI. It provides diverse information for the clinical doctors or researchers, including MI subtypes, risk factors, multi-omics data, sample information (e.g. sample population, gender, region, medical history, etc.), application information, etc. Therefore, clinicians can search specific factors for personalized diagnosis, treatment and prognosis from MIKB combined with the personal information of MI patients. (2) MIKB can provide concepts for building MI ontology in the future, and it is of great significance to realize data standardization, break data islands, and share data to promote research for MI. (3) The structured information of MIKB can be transformed into a knowledge graph, and then an intelligent chat system can be developed to guide clinicians, patients and the general population. (4) Clinicians and researchers can quickly and easily find various factors of the MI subtypes they are interested in by searching MIKB. (5) Through further analysis of the relationship between multi-omics factors and diverse MI subtypes, and then specific molecules of MI subtypes can be found for personalized medicine. Consider MI as a picture and a large number of MI studies as the fragments of the picture. Although each fragment of the picture is important, it is impossible to construct the picture from one of the fragments. What MIKB does is to collect these fragments to describe the whole appearance of MI for system-level understanding and heterogeneity research.

## 4 Conclusion

MIKB is the first specific and manually curated knowledge base for MI, which is the updated and expanded version of MIRKB. In this version, it not only includes MI-related risk factors and risk models, it also includes the knowledge about the MI-related genes collected from various omics levels. MIKB is an important resource for MI research, providing clinicians a systematic view for the heterogeneous MI. We will keep it improved and updated for the future translational research on MI.

## 5 Data availability

MIKB is freely available at http://www.sysbio.org.cn/mikb/.

## Author contributions

BS and CZ conceived and designed the study; CZ drafted the manuscript; BS and YZ edited the final manuscript; CZ, YZ, RW, KZ and WS performed text mining, data extraction and data annotation; LS, KS, XF and FY checked the data; CZ and XL designed the website. All authors read and approved the final manuscript.

## CRediT authorship contribution statement

**Chaoying zhan:** Data curation, Visualization, Methodology, Writing – original draft. **Yingbo Zhang:** Formal analysis, Writing – review & editing. **Xingyun Liu:**: Visualizations, Investigation. **Rongrong Wu:** Formal analysis, Data curation. **Ke Zhang:** Data curation, Investigation. **Wenjing Shi:** Data curation. **Li Shen:** Data curation. **Ke Shen:** Data curation. **Xuemeng Fan:** Data curation. **Fei Ye:** Data curation. **Bairong Shen:** Conceptualization, Funding acquisition, Supervision, Writing – review & editing.

## Declaration of Competing Interest

The authors declare that they have no known competing financial interests or personal relationships that could have appeared to influence the work reported in this paper.
